# Progress toward new function and design of extracellular G protein-coupled receptor nanobodies

**DOI:** 10.1016/j.molpha.2025.100054

**Published:** 2025-06-20

**Authors:** Roman R. Schlimgen, Brian F. Volkman

**Affiliations:** 1 Department of Biochemistry, Medical College of Wisconsin, Milwaukee, Wisconsin; 2 Program in Chemical Biology, Medical College of Wisconsin, Milwaukee, Wisconsin; 3 Center for Immunology, Medical College of Wisconsin, Milwaukee, Wisconsin

**Keywords:** Nanobody, Variable domain heavy chain–only antibodies, Single domain antibodies, G protein–coupled receptor, Extracellular

## Abstract

Antibodies have played a pivotal role in G protein–coupled receptor (GPCR) research and drug development. Nanobodies, or variable domain heavy chain–only antibodies, have emerged as a next-generation antibody with unique advantages in targeting GPCRs. The first generation of intracellular nanobodies have been instrumental in stabilizing GPCR structures for crystallography and in enabling in vitro GPCR imaging. More recently, extracellular-targeted nanobodies have demonstrated diverse pharmacological profiles, with the ability to modulate GPCR activity, localization, and downstream signaling. With these newly uncovered functional properties, nanobodies can be viewed not only as structural tools but also as modulators of receptor pharmacology. We highlight recent innovations in extracellular GPCR-targeting nanobodies and assess several approaches to accelerate their development as versatile research tools and therapeutics.

**Significance Statement:**

Nanobodies have emerged as a next-generation antibody platform with distinct advantages for targeting G protein-coupled receptors. This review highlights recent advances in extracellular G protein-coupled receptor-targeting nanobodies and explores innovative strategies to accelerate their development as powerful research tools and therapeutic agents.

## 1 Introduction

Monoclonal antibodies have aided in advancing our understanding of G protein–coupled receptors (GPCRs) since as far back as 1973. These first antibodies, designed to target the cell membrane, enabled the visualization and quantification of the fluidity of membranes and their embedded proteins.^1^ With the development of hybridoma technologies in 1975, antibodies could be produced indefinitely in vitro.^2^ This technique rapidly expanded the antibody field, and new antibodies were created that were capable of directly targeting individual GPCRs.^3^ Over the next 2 decades, GPCR-selective antibodies became crucial research tools, facilitating new techniques in the field such as ELISA-based techniques, immunohistochemistry, flow cytometry, and western blotting—ultimately shaping our current understanding of GPCRs.^4^, ^5^, ^6^, ^7^

In the last 5 years, GPCR-targeting antibodies have expanded beyond research tools to become successful therapeutics.^8^ Three antibodies have been US Food and Drug Administration-approved to target GPCRs: erenumab, which targets CGRP-R, mogamulizumab (CCR4), and talquetamab (GPRC5D).^8^, ^9^, ^10^ Although antibodies currently represent a rare class (<1%) of drugs available to target GPCRs, their enhanced selectivity compared with small molecules has led to a rise in antibody-based drug development.^11^^,^^12^ This trend is most dramatic in the chemokine receptor (CKR) subfamily of GPCRs, where high ligand promiscuity and sequence similarity have made small-molecule targeting challenging. For these receptors, the number of approved antibodies now matches that of small molecules over the past 15 years, and over 40% of CKR-targeting drugs currently in clinical trials are antibodies/nanobodies.^9^^,^^12^ However, despite progress in this area, overall, the GPCR field lags in therapeutic antibody development. Although biologics encompass ∼50% of all new Food and Drug Administration approvals, they constitute less than 10% of new approvals targeting GPCRs.^13^ The limited success of antibodies as GPCR drugs is largely due to the tractability of small molecules as well as the receptors’ dynamic, grooved structure, which disfavors antibody binding.^14^, ^15^, ^16^

Variable domain heavy chain–only antibodies (VHHs), also known as nanobodies, are “next-generation” antibodies poised to overcome the challenges of traditional, monoclonal antibodies while improving antibody tractability.^17^ Nanobodies targeting GPCRs have been increasingly used since their pivotal role in stabilizing the intracellular surface of the *β*2 adrenergic receptor structure, leading to the first human active GPCR structures and helping to win the 2012 Nobel Prize.^18^ Since 2012, the development of nanobodies targeting GPCRs has continued, primarily focused on intracellular targeting nanobodies to facilitate further GPCR structure determination and to enable visualization of GPCR localization through microscopy.^19^

Now, just as antibodies transitioned to become GPCR therapeutics, new extracellular-targeting nanobodies have the opportunity to do the same.^20^ In this review, we touch on the structural features of nanobodies, the properties that allow them to overcome pitfalls of traditional antibodies, and dig deeper into the innovative research that is being done to expand nanobodies as GPCR tools and therapeutics. We also discuss new avenues of nanobody development and their implications to accelerate nanobodies as research tools and create a new generation of selective GPCR drugs.

## 2 Results

### 2.1 Antibody versus nanobody: structure and paratope properties

**Fig. 1:**
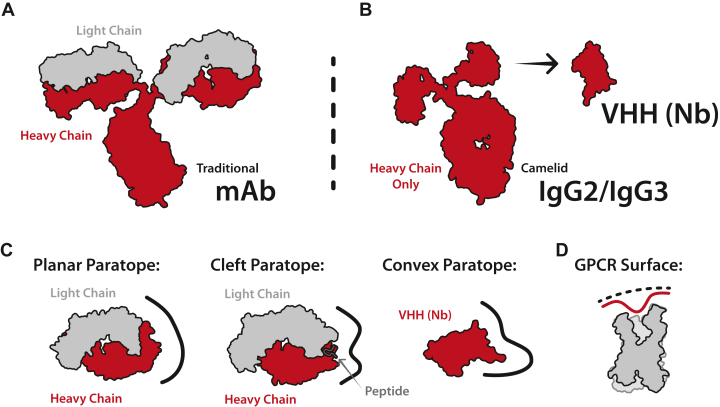
Antibody and nanobody structure and properties. (A) Structural representation of a monoclonal antibody indicating the 2 heavy chains (red) and 2 light chains (gray). (B) Structural representation of a heavy chain–only antibody which is found in camelids in addition to the traditional monoclonal antibody. The variable domain may be removed and used as a small functional antibody (VHH/nanobody). (C) Comparison of the paratope diversity between antibodies and nanobodies. Antibodies often target antigens with planar or cleft paratopes. Nanobodies often utilize planar or convex paratopes to target antigens. (D) Surface illustration of a GPCR, split in half to show the surface areas available for targeting by an antibody/nanobody.

The basic features of immunoglobulin structure were first described in 1959 by Rodney Porter and Gerald Edelman.^21^^,^^22^ Porter cleaved rabbit gamma globulin into 3 components of identical weight and described a constant crystallizable fragment (Fc) and 2 antigen-binding fragments (Fab). He hypothesized that these components assembled into a “Y-like” structure.^21^ Independently, Edelman reduced the intermolecular disulfides of gamma globulin to identify a 50 kDa “heavy” chain and a 25 kDa “light” chain. He reasoned that 2 of each were crucial to assemble the intact 150 kDa antibody molecule.^22^^,^^23^ The data from both scientists successfully described the antibody architecture, earning them a Nobel Prize in 1972.^24^ Although crystal structures of the Fab fragment (Fab) solved in the late 1980s revealed the conserved features of the individual Ig fold domains, the high-resolution structure of an intact antibody was not experimentally determined until 1997 (Fig. 1A, PDBID 1IGT).^25^ Subsequent research on antibodies revealed that the N-terminal Ig domains of the heavy and light chains are the only variable regions of the antibody structure. The antigen-binding properties of antibodies arise from 3 short loops within each variable domain, known as complementary determining regions (CDRs). The CDRs for a particular antibody are specified during B-cell development, when random recombination of variable (V), diversity (D), and joining (J) gene segments selects from a vast repertoire of possible antigen-binding sites. Thus, V(D)J recombination provides the adaptive immune system with a mechanism for the maturation of antibodies that bind antigens with high affinity.^26^, ^27^, ^28^

Many additional antibody isotypes have been characterized in addition to the IgG first described by Porter and Edelman, including IgAs, IgDs, IgEs, and IgMs, all of which include slight variations of the same light and heavy chain architecture.^29^ This was the case until 1993, when researchers in Brussels discovered that llamas have heavy chain–only antibodies in addition to traditional antibodies.^30^ These heavy chain–only antibodies contained an Fc region identical to the human Fc, with a single variable Ig domain in place of the 4 Ig domains that comprise a 50 kDa Fab (Fig. 1B). The absence of the dimeric variable domain enabled the rapid isolation of a monomeric functional domain known as a VHH, or “nanobody” due to its small (∼13 kDa) size.^30^ Since the discovery of these unique antibodies, similar heavy chain–only antibodies have been documented in all camelids (llama, alpaca, camels, etc.), as well as a few cartilaginous fish (sharks, skates, rays, etc.).^30^, ^31^, ^32^

Why camelids and these cartilaginous fish convergently evolved to produce similar heavy chain–only antibodies is not fully understood. However, one of the most reasoned pressures for this evolution is to offer diversity in antibody-antigen interactions.^33^ Traditional antibody binding regions (paratopes) are usually flat to interact with the surface of a globular protein antigen or form a cleft between their heavy and light chains to enfold peptides or other small antigens (Fig. 1C, left, middle).^34^^,^^35^ Nanobodies are more likely to form convex paratopes to access grooves or clefts on an antigen surface (Fig. 1C, right).^14^ Recent work suggests that nanobodies may be more effective than antibodies at binding GPCRs using a convex epitope to target deep transducer-binding or orthosteric GPCR pockets (Fig. 1D).^36^^,^^37^

### 2.2 Intracellular nanobodies to stabilize structure and image GPCRs

**Fig. 2:**
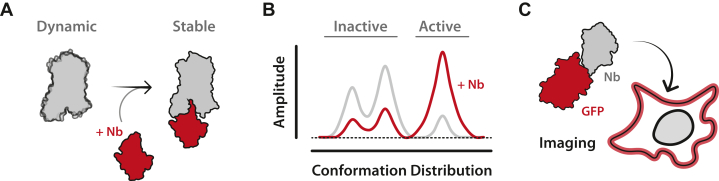
Intracellular nanobodies stabilize GPCR structure and enable visualization of GPCR localization. (A) Illustration of how intracellular nanobodies stabilize GPCR basal dynamics. Nanobody-induced GPCR stabilization has enabled the determination of GPCR crystallographic and cryo-EM structures. (B) A representative plot of conformational distribution is shown with and without a GPCR-targeting nanobody. Biophysical methods such as electron paramagnetic resonance (EPR), nuclear magnetic resonance (NMR), and single molecule FRET (smFRET) provide this information. (C) Cartoon of an intracellular nanobody tethered to a fluorescent protein. Fluorescent nanobody fusions are used to visualize GPCR signaling and localization.

Nanobodies were first introduced into the GPCR field to replicate their use as crystallographic chaperones (stabilizing structural heterogeneity) as demonstrated in other fields by Jan Steyaert.^38^, ^39^, ^40^ With Steyaert’s assistance, in 2011 the lab of Brian Kobilka immunized a llama with pure β_2_AR complexed with a high-affinity ligand and successfully obtained nanobody clones. The lead nanobody candidate (Nb80) stabilized the GPCR active state, demonstrated by a 100-fold enhancement in agonist potency in the presence of the intracellular-binding nanobody, mimicking the native heterotrimeric G protein (Fig. 2A). This nanobody enabled the first active-state structure of a human GPCR.^18^^,^^41^ Shortly after, a new nanobody (Nb35) was developed as another crucial GPCR crystallographic chaperone—although it did not bind a GPCR directly. Instead, Nb35 targeted the interface of the G*α* and G*β* subunits of the heterotrimeric G protein, locking the G protein in its active, receptor bound conformation. This stabilization resulted in another groundbreaking *β*_2_AR structure—now in complex with an active heterotrimeric G protein.^42^ Since then, Nb80, Nb35, and several later developed GPCR-targeted nanobodies have become a staple of GPCR structural studies, resulting in more than 50 new structures of GPCR complexes (Table 1).^36^^,^^43^

**Table 1:** Nanobody-bound GPCR structures

GPCR	Nanobody	Epitope	PDBID
ADRA1A	Nb6	Intracellular	7YMJ, 8HN1
ADRA1A	Nb29	Extracellular	7YM8, 7YMH
AGTR1	Nb.AT110i1	Intracellular	6DO1, 6OS0, 6OS1
AGTR1	Nb206	Extracellular	9EAI, 9EAJ
AGTR1	Nb209	Extracellular	9EAH
AGTR1	AT118-H	Extracellular	8TH3
AGTR1	AT118-L	Extracellular	8TH4
APLNR	JN241	Extracellular	6KNM
B1AR	Nb80	Intracellular	6H7J, 6IBL
B1AR	Nb6B9	Intracellular	6H7L, 6H7M, 6H7N, 6H7O, 7BTS, 7BU6, 7BU7
B2AR	Nb6B9	Intracellular	4LDE, 4LDL, 4LDO, 4QKX, 6N48
B2AR	Nb80	Intracellular	3P0G
B2AR	Nb60	Intracellular	5JQH
B2AR	Nb71	Intracellular	6MXT
CaSR	NB2D11	Extracellular	7E6U
CNR1	CNb36	Intracellular	9B9Y, 9B9Z, 9BA0
CXCR3	Nb6	Intracellular	8HNN, 8K2W
CXCR4	Nb6	Intracellular	8ZPL, 8ZPM, 8ZPN
DRD1	NbA3	Intracellular	8JXR, 8JXS
FZD3	Nb9	Intracellular	8QW4
FZD3	Nb8	Extracellular	8Q7O
LRG4	Nb52	Extracellular	8XFP, 8XFT, 8Y69
M2AR	Nb9-8	Intracellular	4MQS, 4MQT
MC4R	pN162	Extracellular	8QJ2
GRM1	Nb43	Extracellular	7DGE
GRM2	DN13	Extracellular	7EPB, 7E9G
GRM5	Nb43	Extracellular	6N50, 6N51, 8T7H, 8T8M, 8TAO
NTSR1	Nb6	Intracellular	7UL2
OPRM1	Mb6	Intracellular	7UL4
OPRM1	Nb6M	Intracellular	7UL3, 9BJK
OPRM1	NbE	Extracellular	8QOT
ORPK1	Nb39	Intracellular	5C1M, 6B73
ORPK1	Nb6	Intracellular	6VI4
ORPK1	Nb6M	Intracellular	9MQI, 9MQK
OX2R	Sb51	Extracellular	7L1V
RHO	Nb2	Extracellular	8FD0
SMO	NbSmo8	Intracellular	6O3C
SSTR2	Nb6	Intracellular	7UL5
SUCNR1	Nb6	Intracellular	6IBB, 6RNK, 6Z10
US28	Nb7	Intracellular	4XT1, 5WB1, 5WB2

Nanobody utility has rapidly expanded beyond their initial use as crystallographic chaperones. Nb80 and other nanobodies have been used in biophysical studies of GPCRs, providing experimental evidence that GPCRs are not merely “on” or “off” but exist as an ensemble of different conformational states^44^ (Fig. 2B). Nanobodies have enabled real-time measurements of conformational exchange and helped define the molecular switches that govern receptor activation and signaling.^45^, ^46^, ^47^ Beyond conformational dynamics, nanobodies have helped to reveal the spatial complexity of GPCR signaling. By modifying nanobodies for intracellular expression and tagging them with fluorescent proteins, the lab of Mark von Zastrow demonstrated that GPCRs continue to signal from intracellular compartments such as endosomes, challenging the canonical view of plasma membrane-restricted signaling (Fig. 2C).^48^^,^^49^ This foundational work was extended shortly after by the Lefkowitz lab, which developed a suite of conformation-specific nanobody biosensors that allowed real-time tracking of GPCR activation states and demonstrated that distinct signaling outcomes can be linked to receptor location.^50^ Collectively, these studies and many others have demonstrated the importance of nanobody-based biosensors as tools for dissecting GPCR trafficking, internalization, and recycling in live cells.^51^^,^^52^ In a short time, nanobodies have become indispensable tools in GPCR biology.

### 2.3 Extracellular GPCR-targeting nanobodies

Most of the originally developed GPCR nanobodies targeted intracellular epitopes and are often referred to as “intrabodies.”^53^ The disproportionate targeting of intracellular epitopes over extracellular epitopes was thought to have arisen due to many inherent obstacles in the immunization process. Early immunization was conducted with the extracellular pocket occupied by a small molecule, which disfavored interactions at this site.^18^ Later, it was recognized that immunization tended to enrich for intracellular epitopes regardless of the presence of small molecules, likely due to their greater dissimilarity to the exposed surfaces of llama GPCRs and the larger surface area of intracellular GPCR epitopes. Improved screening methods and immunization with GPCRs with shrouded intracellular surfaces have increased the availability of extracellular nanobodies.^36^^,^^54^^,^^55^ Consequently, recent studies have employed extracellular nanobodies, which can modulate GPCR activity, localization, and downstream signaling pathways, offering new avenues for therapeutic intervention and new insights into GPCR biology (Table 2). Extracellular-targeting nanobodies can be divided into 2 categories based on their GPCR epitopes: competitive (orthosteric) nanobodies and noncompetitive nanobodies.

**Table 2:** Nanobodies with extracellular GPCR epitopes

GPCR	Nanobody	Epitope	Extracellular Function
ACKR3	NB1-5, VUN701	Orthosteric	Antagonist
ACKR3	VUN700, VUN702	Orthosteric	Inverse Agonist
ADRA1A	Nb29	Allosteric	PAM
ADGRG2	Nb23-bi	Allosteric	PAM
AGTR1	AT118, AT118-H, AT118-L, AT206, AT209	Orthosteric	Antagonist/NAM
AGTR1	AT118i4	Orthosteric	Inverse Agonist
APLNR	JN241	Orthosteric	Antagonist
APLNR	JN241-9	Orthosteric	Agonist
CaSR	NB2D11, NB32, NB88	Allosteric	NAM
CMKLR1	CA4910, CA5183	Orthosteric	Antagonist
CX3CR1	54A12, 54D05, 66B02, 66G01, BI655088, BI655089	Orthosteric	Antagonist
CXCR2	2B2, 127D1, 54B12, 97A9, 163E3, 163D2	Orthosteric	Antagonist
CXCR4	238D2, 238D4, 10A10, VUN400-VUN410	Orthosteric	Antagonist
FZD3	Nb8	Allosteric	NAM
GLP1R	Nb GLP1R	Extracellular	Altered Ligand Selectivity
GRM2	DN1, DN10, DN13	Allosteric	PAM
GRM4	DN42, DN45	Allosteric	PAM
GRM5	Nb43	Allosteric	NAM
LRG4	Nb52	Allosteric	Unpublished
M1R	NbF3, NbF7, NbA12	Orthosteric	Antagonist
M1R	Nb1B4	Orthosteric	Partial Agonist/PAM
MC4R	pN162	Orthosteric	Agonist
MRGPRX2	Sim4784, Sim8619, Sim9877	Orthosteric	Antagonist
OPRM1	NbE	Orthosteric	Antagonist
OX2R	Sb51	Allosteric	Structure Determination
PTHR1	Nb PTHR1	Extracellular	Altered Ligand Selectivity
RHO	Nb2	Allosteric	PAM
US28	US28-NB	Orthosteric	Antagonist

#### 2.3.1 Competitive nanobodies

**Fig. 3:**
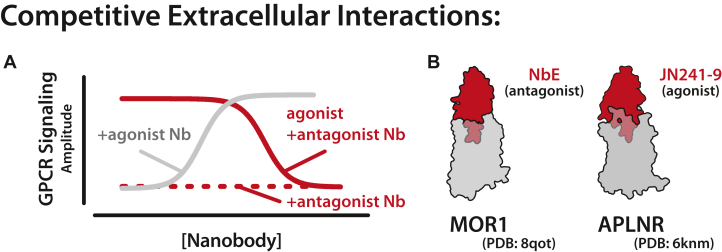
Pharmacology of competitive GPCR nanobodies. (A) Representative signaling assay showing competitive nanobody pharmacology. Most competitive nanobodies act as GPCR antagonist (red) and prevent GPCR signaling. JN241-Y acts as a competitive agonist (gray) and stimulates GPCR signaling. (B) Structure of the antagonist NbE-MOR complex (left) and the agonist JN241-Y-APLNR complex (right).

Nanobodies that compete directly with endogenous ligands for the orthosteric site of a GPCR are viewed as the most promising candidates for clinical applications. To date, nearly all competitive nanobodies elicited by camelid immunization are antagonists, binding to the orthosteric site of their GPCR and preventing signaling by endogenous ligands (Fig. 3A). Examples include nanobodies targeting CXCR2 (2B2), CXCR4 (VUN400), ACKR3 (VUN701), APLNR (JN241), AT1R (AT118i4), and MOR1 (NbE) (Fig. 3B, left).^47^^,^^54^^,^^56^, ^57^, ^58^, ^59^, ^60^ The tendency to generate antagonist nanobodies may again be due to the design of camelid immunization. For the development of each nanobody listed here, camelids were administered an inactive, apo-state GPCR, favoring the generation of nanobodies to target this inactive state. Supporting this hypothesis, the MC4R nanobody pN162 is currently the sole agonist nanobody generated through camelid immunization and was elicited using an MC4R locked in an active conformation by an intracellular nanobody, Cb80.^61^

Despite this limited pharmacological diversity, it has now been demonstrated that nanobody pharmacology can be tuned with simple modifications. Bivalent nanobodies often demonstrate inverse agonism, and CDR sequence changes can convert an antagonist to an agonist as demonstrated by Ma et al (Fig. 3A).^43^^,^^62^ In this example, a tyrosine insertion in CDR3 of JN241 created an apelin receptor agonist (JN241-Y) that promotes receptor signaling (Fig. 3B, right).^58^ Regardless of their pharmacological profile, all current competitive nanobody structures use a convex paratope in CDR3 to facilitate orthosteric interactions, something implausible for traditional monoclonal antibodies (Fig. 1C).

#### 2.3.2 Noncompetitive nanobodies

Nanobodies that do not directly compete for the orthosteric site may appear less significant, but innovative research has shown that these nanobodies have a wide range of functions that are valuable in both GPCR research and therapeutics.

**Fig. 4:**
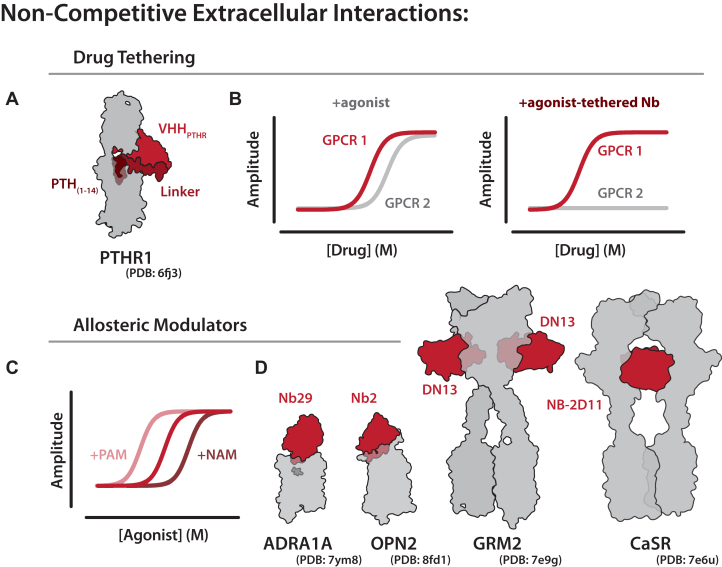
Pharmacology of noncompetitive GPCR nanobodies. (A) Cartoon of PTHR1 bound to a nanobody (VHH_PTHR_) c-terminally linked with PTH. (B) Representative signaling assay showing a nonselective agonist for 2 GPCRs and the same nonselective agonist activity when tethered to a selective nanobody. (C) Dose-response curve for an agonist (red) and an agonist in the presence of a positive allosteric modulator (PAM, light red) or negative allosteric modulator (NAM, dark red). PAMs and NAMs can also result in an increase or decrease in the signaling efficacy, respectively. (D) Structure of 3 PAM nanobodies Nb29, Nb2, and DN13 (left) bound to their cognate GPCRs. Structure of a NAM nanobody NB-2D11 bound to CaSR.

In 2020, Ross Cheloha recognized that noncompetitive nanobodies targeting the extracellular domain of the parathyroid hormone receptor 1 (PTHR1) exhibited a much higher selectivity than the endogenous parathyroid hormone (PTH), which binds both PTHR1 and PTHR2. By tethering the C-terminus of the selective PTHR1 nanobody (VHH_PTHR_) to the PTH C-terminus, he created a drug-tethered nanobody that demonstrated agonism favoring PTHR1 (Fig. 4A).^63^^,^^64^ In this way, conjugated nanobodies can be used to increase exogenous drug potency for a given GPCR while diminishing the potential of off-target effects (Fig. 4B). Moreover, by coupling these noncompetitive nanobodies to a range of different ligands, this approach may also enable the selective engagement of specific receptor conformations and signaling pathways. For example, the peptide PTH_(1–11)_, which is normally a balanced agonist at PTHR1, was converted into a highly biased agonist favoring G protein signaling when tethered to VHH_PTHR_. This work and subsequent studies have shown that nanobody-ligand conjugates can reprogram the signaling profile of otherwise unbiased or suboptimal ligands, offering a powerful strategy to fine-tune GPCR pharmacology for both basic research and therapeutic applications.^64^, ^65^, ^66^

A special type of noncompetitive interaction is an allosteric modulator, which can influence GPCR signaling without directly binding to the traditional ligand-binding site. Nanobodies are increasingly demonstrated as effective allosteric modulators, most commonly as positive allosteric modulators, which enhance the potency or efficacy of signaling responses without directly competing with the endogenous ligand (Fig. 4C).^67^ For instance, Nb29 acts as a positive allosteric modulator, binding the active conformation of ADRA1A and capping the orthosteric site to trap small molecules inside the binding site (Fig. 4D). This nanobody binding enhances the potency of orthosteric ligands.^68^ Similar to Nb29, Nb2 binding to the extracellular loops and N-terminus of rhodopsin can decrease the rate at which the receptor returns to the ground state, positively modulating photoactivation.^69^ In class C GPCRs such as mGlu2, the nanobody DN13 stabilizes the active dimer interface, reducing receptor recycling and enhancing receptor signaling.^70^^,^^71^

Conversely, some nanobodies function as negative allosteric modulators by interfering with receptor activation without directly competing at the orthosteric site. For example, the CaSR-specific nanobody Nb32 inhibits signaling by blocking a dimerization interface that is required for activation.^67^^,^^72^ Notably, recent work from the Kruse lab demonstrated that these described allosteric effects can be tuned through rational nanobody engineering or selection. In this study, nanobody AT118, which targets the angiotensin II receptor type 1 (AT1R), was modified to either promote or hinder the binding of specific ligands by selectively sterically blocking access to the orthosteric site. These engineered variants exhibited distinct effects on ligand engagement, demonstrating that in the future, nanobody design could be used to modulate ligand bias and tailor unique signaling outcomes.^55^^,^^73^

Together, these innovations highlight the versatility of noncompetitive nanobodies, and demonstrate the unique ability of the molecules to fine-tune GPCR activity in ways that may be inaccessible to traditional small molecules.

### 2.4 Nanobody development

#### 2.4.1 Camelid immunization and screening

**Fig. 5:**
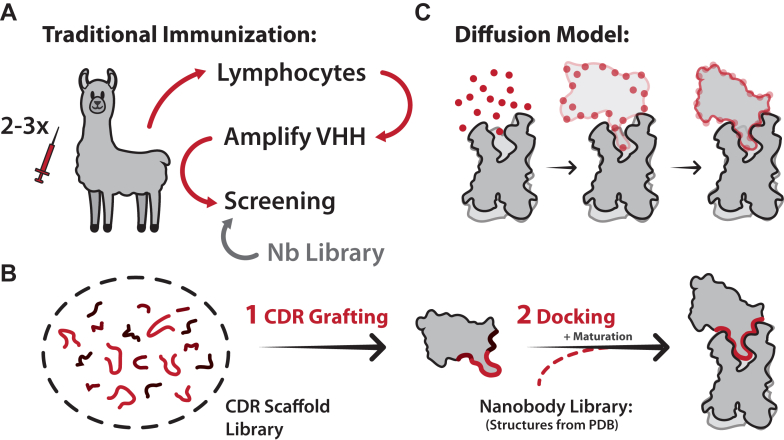
Experimental and computational methods for nanobody development. (A) Overview of the traditional camelid immunization workflow to generate nanobodies. Camelids are immunized multiple times with purified antigens. After several weeks, a small amount of blood is drawn, and lymphocytes are isolated. VHHs are amplified from the camelids and screened to find the best hits. Optionally, naive or synthetic nanobody libraries can be used to begin nanobody development at the screening step. (B) Computational methods are also used to develop nanobodies. The most common technique is “CDR Grafting” which uses a library of CDR scaffolds to create nanobodies. Synthetic nanobodies are then screened for binding with molecular docking techniques. Docking itself can also be utilized to screen for nanobody hits often followed by affinity maturation experiments. (C) A new computational approach for nanobody design uses machine learning trained on antibody-antigen interactions to produce nanobodies against a target epitope. In this method, random noise is iteratively transformed into a nanobody structure that resembles the training dataset.

Nanobody development is a multistep process beginning with the immunization of a camelid (usually a llama) multiple times by a purified protein antigen. After several weeks, a small amount of blood is drawn to isolate the B cells, which are used to amplify the VHH. Amplified nanobodies undergo phage display, cloning of positive hits, purification, and further pharmacological screening.^74^ This entire immunization and screening process can take between 6 months and 2 years to identify and characterize a high-affinity nanobody clone (Fig. 5A). This immunization process can be explored in more detail in reviews.^74^^,^^75^

To bypass the bottleneck of animal immunization, alternative strategies using naive camelid nanobody libraries and synthetic nanobody libraries enable the development of lead nanobodies from the screening stage (Fig. 5A). In particular, synthetic nanobody libraries have become one of the most favorable techniques due to their rapid turnaround and tailored designs, which include libraries that are optimized based on structural databases or that bias hits toward specific nanobody CDR3 conformations.^76^^,^^77^ When high-quality libraries are accessible, binders can be identified in just weeks through yeast, phage, and ribosome display platforms, representing a major advantage over traditional immunization timelines. However, despite the advantages, these libraries come with their own challenges: access is often restricted, library quality varies widely, and selected hits frequently exhibit polyreactivity, poor biophysical properties, or reduced thermostability relative to their in vivo-matured counterparts. ^78^ As a result, development timelines can be significantly longer than published expectations.

Despite the effectiveness and routine use of both immunization and library screening, these processes can introduce biases that can benefit or hinder nanobody development. Copurification of *β*_2_AR with the agonist BI-167107 led to the beneficial generation of a conformational stabilizing Nb80 that mimicked an endogenous G protein.^18^ However, this example also highlights a key challenge in nanobody discovery: targeting active extracellular GPCR conformations remains inherently difficult. Agonist binding is typically required to promote these conformations, which can obscure key extracellular epitopes and limit access to functionally relevant microswitch regions. To address this, emerging strategies have shifted toward the use of stabilized or “locked” receptor constructs that mimic specific signaling states in the absence of ligand. These engineered GPCRs enhance epitope accessibility and improve selection specificity, enabling the identification of nanobodies that discriminate between active and inactive conformations. This approach has recently proven successful for the melanocortin 4 receptor (MC4R) and muscarinic acetylcholine receptor M1 (M1R), where traditional immunization strategies would likely be ineffective.^61^^,^^79^

#### 2.4.2 Computational nanobody design

Computational design has emerged as a powerful alternative to experimental nanobody discovery, offering the potential to accelerate timelines, reduce cost, and overcome some of the inherent limitations of animal immunization and library screening. Methods to computationally generate antibodies have been around since 2007, the most prominent being “CDR grafting.” ^80^ CDR grafting involves iteratively replacing the CDRs of an antibody with a library of structurally diverse loops derived from known antibody structures. Each designed variant is then docked against a static model of the antigen, and the resulting interface is evaluated using scoring functions that approximate binding affinity and stability (Fig. 5B).^81^^,^^82^ CDR grafting has since been extended to nanobody development, and a number of computational tools—including RosettaAntibodyDesign,^83^ OptCDR,^84^ AbDesign,^85^ and OptMAVEn^86^—have been developed to automate this process, with several reported successes in generating nanobodies with high-affinity binding. A key strength of this approach (and of computational design more broadly) is the ability to target specific conformational states of an antigen, bypassing the selection bias of dynamic immunization by using a static structural model as input.

The large number of high-resolution antibody and nanobody structures has also enabled structure-based nanobody docking itself to become a useful computational approach.^87^ Analogous to small-molecule virtual screening, this approach docks experimentally determined nanobody structures against a static model of the target antigen, identifying candidates based on interaction energy (Fig. 5B, right). These hits can then be refined using energy minimization, molecular dynamics, or experimental maturation. However, like CDR grafting, this method depends heavily on the diversity and quality of existing structural templates, which can constrain the novelty and conformational specificity of designed nanobodies, particularly when targeting dynamic proteins such as GPCRs, which are often structurally characterized with nanobodies in only a single, typically inactive, state.

The newest and most significant advancement in nanobody design has come from the integration of deep-learning–based structure prediction tools (AlphaFold/RosettaFold) with generative design models, most notably with the development of RFdiffusion.^88^ This platform, based on RosettaFold and enhanced with denoising diffusion probabilistic models, allows for de novo generation of protein binders. RFdiffusion works by introducing stochastic noise into the 3D coordinates of an antigen-bound complex and learning to reverse the noise in a way that produces realistic and high-affinity protein interfaces. Unlike earlier approaches, RFdiffusion does not rely directly on grafting or static structural libraries; instead, it captures features of protein-protein interaction from Protein Data Bank (PDB) derived datasets and applies them to create novel protein backbones. These generated scaffolds are then sequence optimized using tools such as ProteinMPNN, enabling rapid, structure-aware design of nanobodies and other binding proteins (Fig. 5C).^89^^,^^90^

The practical impact of generative design is already becoming evident in GPCR research. The David Baker lab, in collaboration with GPCR-focused investigators, recently applied RFdiffusion to design high-affinity “minibinders” targeting several challenging receptors, including MRGPRX1, CXCR4, GLP1R, GIPR, GCGR, and CGRPR.^91^ Building on this momentum, both the Andrew Kruse lab and Nabla Bio have adapted similar approaches to develop de novo nanobodies against GPCRs such as MRGPRX2, CXCR4, and ACKR3, achieving nanomolar affinities and demonstrating a range of functional activities in receptor signaling assays.^92^^,^^93^ These rapid advances, all within the past year, underscore the incredible potential of deep learning–based design to access previously intractable GPCR conformations and modulate them with tailored pharmacological nanobodies.

Although promising, these approaches still warrant caution. It remains unclear whether generative models trained on current structural datasets will constrain the diversity or novelty of designed nanobodies. At present, camelid immunization remains the most experimentally validated and broadly effective strategy for nanobody discovery, particularly when antigen stability or epitope accessibility is uncertain. However, the integration of generative machine learning models such as RFdiffusion with experimental validation and refinement pipelines is poised to become a defining shift in the field—paving the way for faster, targeted development of nanobodies against even the most elusive GPCR targets.

## 3 Future directions

Nanobodies have rapidly become indispensable tools in advancing our understanding of GPCR biology. These small proteins have proven their utility in determining GPCR structures and tracking receptor signaling localization. With a tunable pharmacological profile, GPCR nanobodies are poised to become an important new class of biologic drugs. However, similar to the early days of antibody research in the 1970s, the full potential of nanobodies in the GPCR field has yet to be realized. With each innovative application, these selective tools gain increasing utilization and respect within the GPCR research community.

In the next few years, we anticipate that nanobodies will continue to solidify their role as essential GPCR research tools. New methods in computational nanobody design, which can bypass the need for purified proteins, detailed structural information, or pre-existing sequence/structure libraries, are poised to accelerate nanobody development and may enable the characterization of understudied, poorly targeted, and orphan GPCRs. Nanobodies have the potential to be not only be outstanding research tools but also, through new, innovative techniques, exceptional therapeutics.

## Conflict of interest

B.F.V. has ownership interest in Protein Foundry, L.L.C. and XLock Biosciences, Inc.
